# ePhysio: A Wearables-Enabled Platform for the Remote Management of Musculoskeletal Diseases

**DOI:** 10.3390/s19010002

**Published:** 2018-12-20

**Authors:** Carlo Vallati, Antonio Virdis, Marco Gesi, Nicola Carbonaro, Alessandro Tognetti

**Affiliations:** 1 Dipartimento di Ingegneria dell’Informazione, University of Pisa, 56123 Pisa, Italy; antonio.virdis@unipi.it (A.V.); nicola.carbonaro@centropiaggio.unipi.it (N.C.); alessandro.tognetti@unipi.it (A.T.); 2 Dipartimento di Ricerca Traslazionale e delle Nuove Tecnologie in Medicina e Chirurgia e Centro Dipartimentale di Medicina Riabilitativa “Sport and Anatomy”, 56123 Pisa, Italy; marco.gesi@med.unipi.it; 3 Centro di Ricerca “E.Piaggio”, 56123 Pisa, Italy

**Keywords:** wearable devices, rehabilitation systems, musculoskeletal diseases

## Abstract

Technology advancements in wireless communication and embedded computing are fostering their evolution from standalone elements to smart objects seamlessly integrated in the broader context of the Internet of Things. In this context, wearable sensors represent the building block for new cyber-physical social systems, which aim at improving the well-being of people by monitoring and measuring their activities and provide an immediate feedback to the users. In this paper, we introduce ePhysio, a large-scale and flexible platform for sensor-assisted physiotherapy and remote management of musculoskeletal diseases. The system leverages networking and computing tools to provide real-time and ubiquitous monitoring of patients. We propose three use cases which differ in scale and context and are characterized by different human interactions: single-user therapy, indoor group therapy, and on-field therapy. For each use case, we identify the social interactions, e.g., between the patient and the physician and between different users and the performance requirements in terms of monitoring frequency, communication, and computation. We then propose three related deployments, highlighting the technologies that can be applied in a real system. Finally, we describe a proof-of-concept implementation, which demonstrates the feasibility of the proposed solution.

## 1. Introduction

Nowadays, wearable sensors are the principal component of a system for monitoring, in an accurate and reliable way, human activities and behaviors. Wearable sensors are used in many applications such as in the biomedical field, sport, and entertainment [1]. Recently, many groups have explored the possibility of using consumer wearable devices for e-Health applications [2,3,4]. People can use wearable systems during their common, daily-life activities, thus enabling the opportunity to retrieve real-time user data continuously and without discomfort and providing new opportunities to self-assess physical activities along with the health status and well-being. 

Recent advancements in wireless technologies and communication protocols [5] are fostering the seamless integration of wearable devices into a larger system, thus promoting their evolution from standalone elements connected to vertical ad-hoc systems to smart objects integrated in the broader context of the Internet of Things (IoT). In this context, wearable embedded systems represent a new building block for the creation of a new generation of cyber-physical system, in which humans are central, along with their data and social interactions. These Cyber-Physical Social Systems (CPSS) [6] are expected to offer a complex architecture in which the physical space, the cyberspace, and the social space are integrated together to enrich humans’ activities. 

One of the expected application fields for CPSS is the medical field. Smart medical services for automatic patient monitoring and diagnosis [7] and dynamic association of medical and ambient information [8] are only examples of future CPSS that could significantly improve the medical field. In this field, the use of wearable devices in combination with dedicated mobile phone applications could make the difference, for instance, in the long-term remote treatment of a chronic disease delivering personalized feedback to the patient and motivational coaching supervised by professionals. This approach has already been proved to be successful for the treatment of several patient conditions such as obesity, diabetes, and Parkinson’s disease [9,10].

In our study, we are facing the problem to overcome the disadvantages of the classical rehabilitation treatment of Musculoskeletal diseases (MSD) by developing a new platform capable of monitoring and leading the rehabilitation task in different user scenarios outside the clinical center. MSDs are defined as a group of inflammatory conditions and degenerative diseases that commonly generate pain and/or limitation of physical function in the patient. MSDs can involve different body parts including the lower back, neck, shoulders, and upper/lower extremities. 

**Figure 1:**
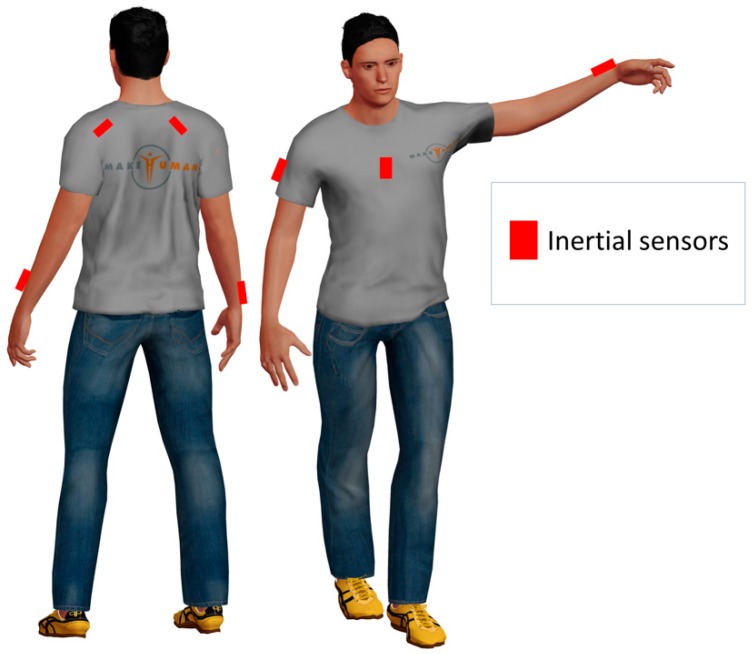
Exemplary application of inertial devices to monitor movements.

As a possible example, one may consider the application of inertial devices to monitor the upper limb movements for shoulder MSD rehabilitation. A possible sensor deployment for this specific case is reported in Figure 1. As we showed in our preliminary work reported in [11], inertial sensors can be placed on the sternum, the forearm, and the scapula.

Physical rehabilitation is a common solution in the therapeutic protocol focused to reduce patient pain and re-establish the correct kinematics of the involved joints in order to restore a functional level of activity. The traditional approach to the foreseen pathology includes physiotherapy sessions and changes in the patient’s lifestyle to avoid degeneration. The repetitive nature of the rehabilitation tasks, the lack of compliance and motivation to perform the training protocol, and finally, the discontinuous interaction with the physician/physiotherapist are the main impediments to the success of such approach.

In this paper, we present ePhysio, a modular sensing platform to monitor, stimulate, and encourage patient activity performance using also gamification strategies and social engagement. Specifically, the system allows the creation of a “virtual link” between patient and clinician (physiotherapist/physician), sending information about the adherence and quality of training protocol, thus providing a real-time feedback to the user regarding correct/incorrect exercise practices. Specifically, we first present a detailed analysis of the use cases to highlight the requirements of the systems and, then, we introduce the overall system architecture and we present a proof-of-concept implementation developed using off-the-shelf hardware to demonstrate the feasibility of our solution.

The rest of this paper is structured as follows: in Section 2 we discuss the background and related work on MSD treatment, whereas Section 3 describes three possible use cases. In Section 4, we detail the ePhysio architecture in terms of its logical organization, the information flow, and the deployment of the three use cases. Section 5 describes a proof-of-concept implementation of the system, with details on the used technologies and examples of user interfaces. Finally, Section 6 concludes the paper and illustrates planned future works.

## 2. Background and Related Work

### 2.1. Definition and Clinical Treatment of MSDs

MSDs are degenerative diseases and inflammatory conditions that concern different body parts such as joints, muscles, ligaments, and tendons. The most common symptom of the MSDs is pain that could be of a different level (from mild to severe), could last for a long period, and could be specific to the interested joint or diffuse in a bigger body area. Persistent pain caused by muscoloskeletal conditions reduce people’s capacity to participate in social roles and, more importantly, limit dexterity and functional ability at work. These conditions will impact on the health status of the patient, involving the loss of participation in the community, decreased quality of life, and mental well-being [12]. The Global Burden of Disease (GBD) report highlights the significant disability burden associated with musculoskeletal conditions. Moreover, this study reports that people having a painful muscolosckeletal condition represent 20–33% of the world’s population, and this pathology is the second major contributor to global disability [13,14]. Another study reports that the number of adult Americans suffering from musculoskeletal conditions represents the same amount of patients with cardiovascular or chronic respiratory diseases [15].

The patients considered here are people of all ages that for work, mainly, or for sport related activities are subject to MSDs due to incorrect posture or repetitive movements executed for a long period of time. Some examples of common MSDs are shoulder impingement (SI), knee osteoarthritis (KOA), lower back pain, and carpal tunnel syndromes. These diseases are considered a primary cause of disability [9].

The treatment program designed by the professional (physiotherapist/physician) consists of different phases; (a) diagnosis: A complete and accurate evaluation of the patient’s status to identify all causative factors; (b) rehabilitation protocol: The program is focused to improve flexibility, mobility, and range of motion (ROM) of the affected joint and to restore muscular balance and neuromuscular control. The clinicians are also relevant during the rehabilitation period in order to check the progress and assess the effectiveness of the training program.

The possibility to perform rehabilitation treatment directly at home is a result achieved by the recent advancements of human motion tracking technologies, i.e., inertial measurement systems (IMUs) that allow the deployment of real-time monitoring data to the clinician and protocol treatment self-management capabilities to the patient. Moreover, the introduction of a solution based on the exergaming capabilities [16], that combines exercise therapy with a human motion sensing system and serious games, have improved the attention of the patient to self-manage their health status following the programs prescribed by rehabilitation professionals.

To date, the system technologies capable of managing the treatment of MSD patients outside the clinical center (i.e., at home) consist of camera-based systems or the integration of wearable sensor devices. Solutions for the recovery of the shoulder impingement disorders have been studied using Microsoft Kinect as camera-based technologies [17,18,19]. 

This kind of solution is convenient when simple therapy is prescribed. In fact, the system is not able to correctly reconstruct complex movements performed by the patient during the exercise therapy. Despite the lack of accuracy, it allows the differentiation of correct movement from wrong ones, and gives reliable feedback to the patient during the rehabilitation session. Moreover, the limitation of the system is mainly based on the biomedical model used for the reconstruction of the shoulder joint that does not correctly describe the complex relationship between the scapular-thoracic and the glenohumeral joints.

Solutions for the treatment of MSDs at home that integrate wearable sensor devices are very limited. A study proposed by Stutz et al. [20] is focused on the recovery of the frozen shoulder using a mobile application. The system gathers information regarding patient exercise activities using the sensors embedded in the smartphone. The digital application guides the patient to perform the correct movements; however, it has not implemented any type of feedback to the patient to modify/adjust the quality of the exercise during its execution. No real-time interaction is foreseen between the patient and the professionals.

### 2.2. Wearable Sensors for Healthcare

Smart wearable devices can detect the wearer or the environment parameters. These data can be stored, transmitted, and elaborated to provide feedback to the user or to other involved players. Wearable interfaces and smart textiles have been developed to enable continuous long-term monitoring of health (heart rate, respiration, and biomarkers) and physical performance (body movements, gait analysis, activity tracking, and rehabilitation) [21,22,23]. To date, the majority of wearable motion monitoring solutions, relevant for our MSD management scenario, rely on multiple tri-axial IMUs applied to different body segments. IMUs estimate the orientation of the body segments at the locations where they are attached by combining multi-sensor information *(tri-axial accelerometer, gyroscope, and magnetometer)* through dedicated optimal sensor fusion algorithms mainly based on Kalman filtering [24]. The combination of different IMUs, placed on connected body segments, and the additional information on the kinematic constraints (biomechanical models) enable the estimation of joint angles. As an alternative, e-textile solutions for wearable motion monitoring have been developed [25,26,27]. Textile-based solutions have the advantages of being low cost, lightweight, of low thickness, flexibility, and the possibility of adaptation to different body structures. By exploiting e-textile sensors, it is possible to design sensing garments where the sensors are “hidden” into the textile of the clothes. Despite these attractive characteristics, textile sensor adoption is still limited, mainly due to the low reliability which limits their use to the reconstruction of large and slow movements. In our recent works, we have proposed hybrid solutions that fuse inertial and e-textile sensors to improve reliability while increasing the wearability of the system [28,29].

It is worth mentioning that recent research works have highlighted the potential security risks connected to the use of smartwatches [30]. Although our work is not specifically focused on smartwatches, some of these security threats may still apply to the proposed system. Considering that analyzing and handling them requires a careful evaluation that is outside the scope of this paper, we leave this aspect for future work.

### 2.3. Contribution

In the context of wearable sensors for healthcare, the ePhysio platform adopts the principles of Cyber-Physical Social Systems to build a self-rehabilitation system that allows patients to connect with a professional. The ePhysio system aims at integrating the patient with his/her rehabilitation activities into an information system by exploiting wearable sensors. This integration provides a link between the patient and the professionals, which can be exploited to ensure the proper monitoring and self-management of the rehabilitation process of musculoskeletal disorders, allowing the supervision/collaboration of professionals and possibly enabling social engagement among patients. Compared with other existing solutions, ePhysio does not focus on a specific pathology. Instead, it offers a general-purpose architecture that can be extended to introduce novel sensors and rehabilitative protocols. Moreover, the platform is designed to adapt to different use-cases and application scenarios that go beyond the execution of exercises at home by a single patient. Finally, to the best of authors’ knowledge, the platform is the first one to include the possibility to exploit social engagement among different patients to improve the efficacy of the treatment. 

## 3. Use Cases 

In this section, we provide an overview of the use cases considered in the design of the ePhysio system. For each use case, we describe the actors involved and the possible features, enabled by novel technological advancements and adopted by ePhysio, that could be exploited to improve the efficacy of the therapy or the training. In the description, we also highlight the potential social interactions that could help to improve the efficacy of the treatments and patients’ well-being.

### 3.1. Single-User Physiotherapy

Self-rehabilitation is an important part of MSDs treatment. After an initial set of rehabilitation sessions with the close aid of a physiotherapist, usually, the patient is prescribed with a list of exercises to be performed at home. The therapy is prescribed by a professional, e.g., a doctor or physiotherapist, in a written form, possibly containing a detailed description of each exercise, i.e., for how long each exercise has to be performed and its frequency. The patient performs the exercises at home autonomously and he/she has to self-assess its quality, i.e., if it is executed correctly. Over time periodic follow-up appointments are usually scheduled to check the progress of the recovery and the status of the disease. If needed, the list of exercises can be updated.

Multiple studies, [20,21,22,23,24,25,26,27,28,29,30,31,32,33,34], highlighted how self-rehabilitation and autonomous quality assessment can lead to poor patient adherence to the treatment and a slow clinical course, due to the absence of continuous monitoring from the professionals. This usually leads to slow recovery time and recidivism of the disease.

Remote monitoring of exercises by means of sensors could allow professionals to closely monitor a patient’s activities and progress. The share of data collected from daily physical activities could also allow physicians to evaluate a disease’s progress in a rapid manner and without scheduling control appointments. Real-time monitoring of the physical activities could also help in providing an instantaneous feedback to the patient as he/she executes the exercise, thus improving the quality of the rehabilitation. In addition to this, the collection of performance information on the exercises could allow the implementation of new gamification techniques, which could help in improving patient’s engagement, for instance by means of virtual competitions among multiple patients.

### 3.2. Indoor Group Therapy

For certain conditions—e.g., ankylosing spondylitis [35], joint replacement [36,37] and, in general, for older populations [38]—group sessions of rehabilitation are often prescribed. Such group sessions performed weekly at a gym can be prescribed in addition to self-rehabilitation or as an alternative to increase the level of commitment of the patients.

In a group session, exercises are first described by the physiotherapist, then the patients repeat the exercise simultaneously. The physiotherapist evaluates the execution of the exercise, either by having an approximate glance at the whole group or by quickly checking patients one by one. The accuracy of the monitoring provided by the physiotherapist depends on the number of patients attending the session, which needs to be relatively small. The progress of each individual patient is evaluated periodically (e.g., monthly) by the physician on a dedicated examination, while during the rehabilitation sessions the feedback of the physiotherapist is absent or limited.

Compared with autonomous therapy, group therapy offers a higher level of engagement that can encourage patients to perform the exercises regularly. Moreover, the presence of a physiotherapist can provide a certain degree of control on the quality of the exercises performed by the patients. However, in large groups, the control is usually limited and not accurate.

In this case, real-time monitoring of the exercise by means of sensors could improve the efficacy of the control of the physiotherapist, which could more easily control a large number of patients. Such data could be made available to physiotherapists in real time to spot patients who require immediate feedback and corrections. In addition to this, the data could be shared with the physicians, which could monitor a patient’s status in between scheduled appointments also and introduce modifications to the therapy, if needed.

### 3.3. Outdoor Activities

The rehabilitation of certain MSDs can allow the patient to carry out outdoor training sessions, e.g., running in a track or a soccer field, activities that can be preferable from the patient’s perspective. Patients are assigned with a training program, whose progress is evaluated by a physiotherapist. Exercises are executed by patients across a large area, while the physiotherapist, who remains at a fixed point, is responsible for checking the progress only at the end of the exercise by measuring biomedical signals, e.g., heart rate, and collecting the feedback of the patient on his/her activity.

In this case, the monitoring of biomedical signals and patient’s performance could improve the accuracy of the control of the physiotherapist, whose evaluation could be based not only on patients’ feedback but also on objective data and measurements. Data collection and analysis could also improve patient’s engagement by means of gamification, e.g., by comparing the results between different patients executing the same exercise.

### 3.4. Requirements

All the use cases are characterized by some limitations, which could be mitigated or solved by using novel technologies. In particular, the following limitation and possible improvements can be highlighted:

- *Exercises are not monitored*—as in the first use case—or they are difficult to monitor due to a large number of patients or due to the distance between the patient and physiotherapist—as in the second and third use cases, respectively.
- There is *no tracking of the performance over time* for each patient. Moreover, using mainly visual monitoring of exercises, the evaluation of each of them is not quantifiable. This makes it hard to verify the progress of a patient or to compare the behavior of different patients suffering similar pathologies.
- There is a *lack of continuous involvement of the physician*. The status of the disease and the rehabilitation progress is evaluated only during periodic appointments. This significantly reduces the possibility of changing inaccurate rehabilitation plans or prescriptions.
- *There is a low engagement of the patient*. Techniques like gamification could be employed to improve his/her engagement and increase the efficacy of the therapy. Moreover, even though in some cases the patients are introduced to environments with many peers with similar pathologies, there is no interaction among them. Such an environment could be exploited to create competitive games and improve overall engagement.

Given the use cases described above and the related limitations, a system that exploits state-of-the-art technologies should:

- Implement a self-evaluation mode in which the patient wears the sensors and the application running in the user interface lead him to the measurement of some biomechanical quantities related to the progression of the disease (e.g., arm abduction and flexion ROM in shoulder impingement; measure the knee adduction moments in knee osteoarthritis patients). When possible, the disease evaluation will be performed adopting accepted clinical scales, e.g., Constant–Murley for the shoulder [39,40]
- Implement a rehabilitation modality—by using a gamified framework—in which the user interface shows to the patient the movement to perform (e.g., arm trajectory in space), evaluates the quality of the exercise (deviation from a reference trajectory), and gives feedback of the error in real time. The exercise characteristics—kind of movements, velocities, repetitions—can be set by the physicians/physiotherapist remotely according to the specific needs of a given patient.

An additional non-mandatory feature is the possibility to monitor the patient movement/activity in daily life by using a reduced set of sensors (e.g., inertial sensors of the smartphone or a smart bracelet). This information could be used to complete the data collected during self-evaluation and rehabilitation and to discover, in the long term, correlations between daily life activity patterns and progression/regression of the disease.

## 4. System Architecture

In this section, we first describe the architecture of the proposed system in terms of its main components and functions, highlighting how they interact and communicate with each other. We then present the flow of information through the system, specifying how information is processed and transformed from raw data to high-level information which can be used by professionals. Finally, we will discuss how the ePhysio platform can be adopted in the three use cases presented in Section 3.

### 4.1. Logical Architecture

**Figure 2:**
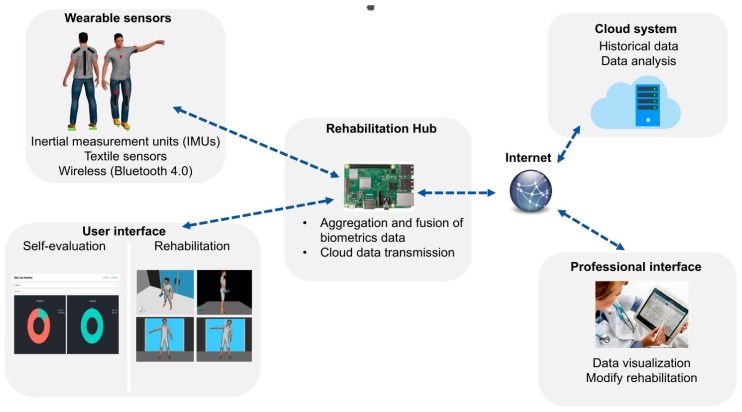
High-level view of the ePhysio architecture.

A high-level view of the logical architecture of ePhysio is shown in Figure 2. A set of wearable sensors is deployed to each patient to monitor their exercises. Such wearable sensors are mainly based on inertial measurement units that can be worn by the patient in an easy and rapid manner, e.g., through removable stripes. Each sensor is equipped with a wireless transceiver to transmit the collected data in real-time, e.g., via Bluetooth, IEEE 802.15.4, or Wi-Fi. Each sensor can either produce raw data or perform a first processing of the obtained measurements, to either refine data or to compress them or both for efficient transmission. Sensors are generally resource-constrained in terms of computational and communication capabilities and they are also battery powered. For this reason, the decision on what communication technology to use and on which operations to perform on the sensors should consider the trade-off between the application requirements and the sensors’ capabilities and battery lifetime.

Data gathered by sensors are sent to a centralized node, called the Rehabilitation Hub (RH), which is the core of the ePhysio system. The RH has a modular structure, which includes two main modules; one for communication and one for data aggregation and fusion. The communication module takes care of implementing the communication functionalities; it is responsible for collecting data from the sensors and for handling the communication with the Cloud System (CS), i.e., to upload the collected data for historical collection and analysis and to receive commands from professionals. Instead, the data aggregation and fusion module implement the post-processing logic required to transform raw data from sensors into refined data. The RH is also connected to a user interface, called the user interface. Such an interface, which can be a screen directly attached or a remote one, is exploited by the RH to show the patient a graphical representation, e.g., through a video of the self-evaluation task or the exercise and a real-time feedback on the accuracy of the execution. To this aim, an initial analysis of the data is performed on the rehabilitation hub to provide a direct feedback to the patient on the execution of the exercise.

The CS that receives the refined data from multiple RHs is responsible to store and organize the data into a database. In addition, for each piece of data, the CS is also responsible for adding the required context metadata, e.g. a patient’s identification and exercise information. Such data is then made available to professionals by means of a web interface, named a professional interface. Such a professional interface is used by physicians and physiotherapists to monitor the correct execution of the exercises and gain an insight into the progress made by the patients, possibly comparing the effectiveness of the exercises of different patient categories. Moreover, the interface allows professionals to provide feedback to patients, e.g., through messages, or alert them as to when it is necessary to change their prescription, e.g., changing the type of exercise or its frequency. In addition to the interface, to aid professionals in the analysis of the collected data, data mining techniques, for instance, based on machine learning or artificial intelligence techniques can be implemented in the platform. These techniques could help professionals to extract high-level behaviors, possibly considering data coming from multiple patients and different time periods.

It is worth mentioning that the ePhysio architecture takes into account the recent research trends in the field of cloud computing architectures. The architecture of the system, comprised of a computational layer for data collection and analysis installed in proximity of the sensors and a cloud computational layer for big-data analysis, is in line with the recent trend of Edge/Fog computing [41]. The latter aims at extending the centralized cloud computing architecture by introducing an additional layer for computation and storage in the proximity of the sensors in order to support applications that require low-latency and resiliency from network outages. The ePhysio architecture based on two levels, i.e., the rehabilitation hub and the cloud system, compared with a direct cloud integration of the sensors, can guarantee the analysis of the data in real-time to provide immediate feedback to the users and also a certain degree of resiliency from network disconnections as data can be buffered to the hub temporarily. Such an approach, also recognized in the literature [42], has some drawbacks considering that eventually the data is always uploaded to the cloud. Although this approach could represent a risk for the security and privacy, especially considering the sensitivity of the data, the cloud backend can ensure a more exhaustive analysis of the collected data, e.g., exploiting the comparison of measurements from different patients. A no-cloud implementation, although possible, was not considered for the ePhysio system as it could significantly reduce the possibility for data analysis and social engagement.

### 4.2. Information Flow

**Figure 3:**
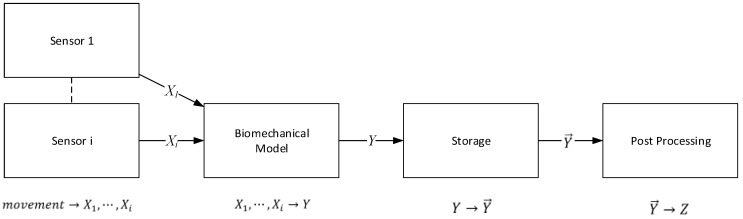
Description of the information flow through the ePhysio system.

As we explained in the previous sections, the purpose of the ePhysio system is to provide remote monitoring and virtual coaching to patients performing rehabilitation tasks outside a clinical center. This means not only gathering and *moving* data from sensors to the various interfaces, both for users and for professionals, but also *transforming* said data from raw data to more refined and enriched versions. This process can be implemented through the information flow summarized in Figure 3. The initial sources of the data are the wearable sensors, responsible for collecting movements and measuring physical parameters. The first level of elaboration can be performed at the sensor level, e.g., considering an IMU, the calculation of the body segment orientation by fusing accelerometer, gyroscope, and magnetometer data. A biomechanical model then combines data coming from multiple sensors applied to connected body segments and produces elaborated data that reconstructs a patient’s movement. For instance, if we consider the biomechanical model of the movement of an arm, the model, implemented in the RH, is responsible for converting the raw data from multiple IMUs into the complete movement of the arm, taking into account the complex evolution of the shoulder. An example of this approach is described in our preliminary works [43,11]. Our model [29] combines the widely used socket-ball with an additional joint, that describes the scapular-thoracic complex and glenohumeral joint and takes into account the constraint given by the scapular-humeral rhythm. The model combines the arm abduction and horizontal flexion angles (detected by arm and sternum IMUs) with the scapular movement (scapular and sternum IMUs) to extract the position of the hand during classical shoulder telerehabilitation exercises and evaluate the difference between the actual and the target arm trajectory. Such refined data is stored into a cloud system along with the associated metadata, e.g., patient’s identification, exercise type, execution time, etc. The data collected from the sensors and the context information can be eventually visualized, for instance as a report of a patient’s performance over time, to professionals. In addition to this, such data can be further analyzed and processed to detect statistical properties, such as anomalies, recurrent errors, etc.

Each elaboration block in the information flow defines only a logical view of the operations that can be executed at each step. The resulting logical architecture can be used to accommodate multiple implementations of each function, tailored to fit the capabilities available at the elements of the architecture, i.e., sensors, rehabilitation hub, etc., and depending on the constraints introduced by the communication links, e.g., limited bandwidth, delay. As an example, in the case that the sensor computation power is limited, the executed biomechanical model can be rather simple, and more complex algorithms can be applied instead within the post-processing. With such an organization of the information flow, the ePhysio system thus provides a flexible architecture.

### 4.3. Deployments

The presented architecture accommodates the functional requirements presented in Section 3.4. However, the three use cases presented in Section 3 are characterized by different specific requirements, both medical and technological, often characterized by tradeoffs. For instance, in order to obtain an accurate monitoring of the exercises, the wearable sensors should be configured to collect measurements with a certain frequency; however, the monitoring frequency is limited by the technology of the sensor, i.e., by its sampling capabilities, its computational capabilities, and its tradeoffs with the sensor’s battery lifetime. In the following subsections, we will analyze three deployments of the ePhysio architecture, one for each of the use cases introduced in Section 3. In particular, for each use case, we analyze the specific functional and technical requirements.

#### 4.3.1. Single-User Physiotherapy

**Figure 4:**
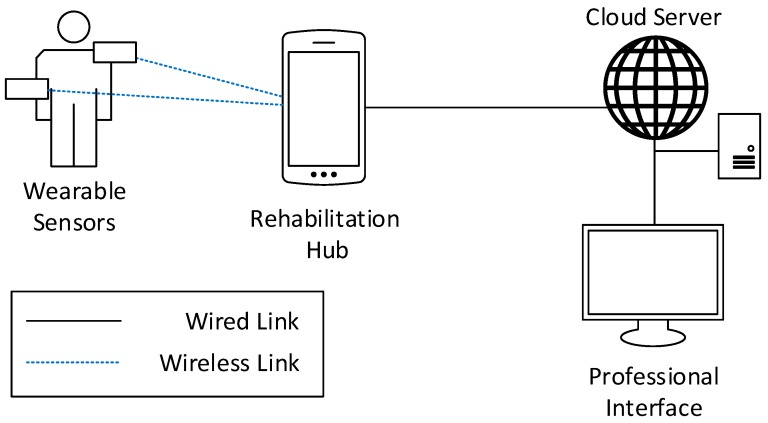
Example of deployment for the single-user physiotherapy use case.

In the single-user use case, Figure 4, the RH is responsible for collecting and processing data from only one patient. For this reason, the RH is not characterized by specific requirements, and could be implemented as a general-purpose device, such as a smartphone or a tablet, for instance by developing an application to collect information from the sensors, elaborate raw data by the application of a specific biomechanical model, and upload them to the CS. The connection between the RH and the sensor can be realized via a short-range wireless link, e.g., Wi-Fi, IEEE 802.15.4, or Bluetooth Low Energy, owing to the short distance between the nodes. The patient interface could be easily implemented, showing on the device screen, the real-time execution of the self-evaluation task or the exercise and some specific parameters useful for the patient to check their performances. Eventually, the dedicated professional interface must allow physicians and physiotherapists to browse information from multiple patients simultaneously.

#### 4.3.2. Indoor Group Therapy

**Figure 5:**
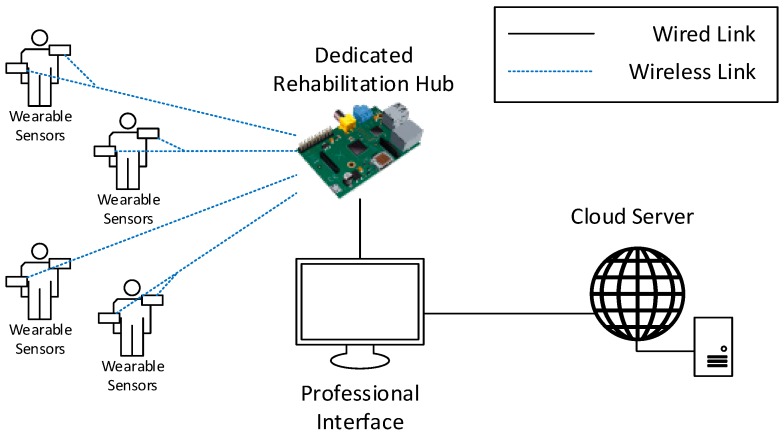
Example of deployment for the indoor group therapy use case.

In indoor group therapy, Figure 5, the system needs to support data collection from multiple patients. The patients could potentially execute different rehabilitation exercises simultaneously. The patients are assumed to perform their activity in the same area, i.e., in direct communication range of a common RH. In order to support the collection of a larger amount of data, the RH in this use case should be implemented as a dedicated board, having sufficient computing and storage capabilities to process the data from all the patients. To this aim, the RH can be implemented as an embedded system on which a specific software service is deployed.

The software should implement the tasks of data collection and data processing, in addition to data offloading to the CS. In order to ensure system expandability and allow rapid and easy integration of exercises for new pathologies and the support for the associated sensors, the software should be implemented with a modular structure.

The RH and the CS should be implemented to allow their interconnection with locally available devices in order to display the patient and professional interfaces to users performing their exercises and to physicians and physiotherapists. For instance, the patient interface could be made available through screens shared among the patients performing the same exercise, while the professional interface could be made available to professionals closely following the progress of groups of patients, for instance, through tablets or laptops.

#### 4.3.3. Outdoor Activities

**Figure 6:**
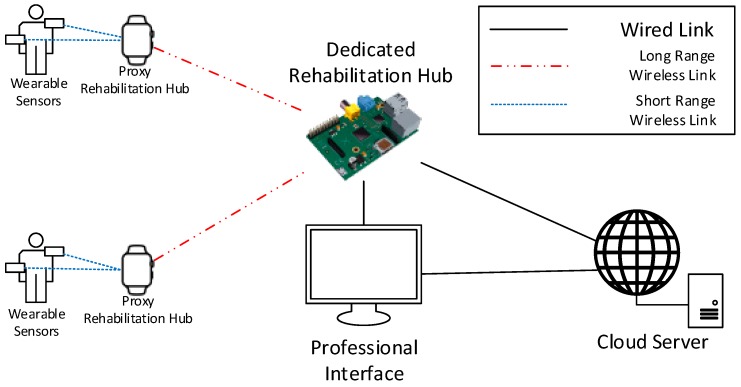
Example of deployment for the outdoor activities use case.

In outdoor therapy, Figure 6, patients are independently performing their exercises in an outdoor environment under the supervision of a professional.

Similar to the indoor therapy use case, the system must support simultaneous data collection from multiple patients potentially executing different exercises. For this reason, the implementation of the RH presents the same requirements of the indoor therapy use case. The only additional requirement is the necessity to cover a larger area compared with the indoor use case. For this reason, a set of proxy RHs can be installed to cover a wider area and relay patients’ data to the RH. The proxy rehabilitation hub could be a wireless signal repeater installed in proximity to the outdoor field or a wearable device like a smartwatch responsible for collecting data from other wearable sensors and forwarding them to the RH. In either case, the proxy RH should be able to maintain a short-range wireless link with sensors, e.g., via 802.15.4, and a long-range one with the RH, e.g., obtained through a cellular connection. Communication modules based on short-range wireless technologies have a lower power consumption with respect to long-range ones; therefore, allowing battery-powered sensors to have longer operational periods.

Eventually, the RH forwards data to the remote CS, which exposes the professional interface made accessible in real-time to professionals on the field, for instance, through tablets or smartphones.

## 5. Proof-of-Concept Implementation

To demonstrate the feasibility of the proposed architecture, a proof-of-concept implementation has been developed with the goal of deploying a prototype with a small subset of functionalities and demonstrating the feasibility of the proposed system. For the sake of convenience, the implementation has been carried out using only off-the-shelf hardware easily available in the market. In the proof-of-concept implementation, we initially focused on deploying a single-user physiotherapy system composed of a set of wearable sensors, a rehabilitation gateway, and a cloud backend to collect data from one single patient and show the data to professionals.

**Figure 7:**
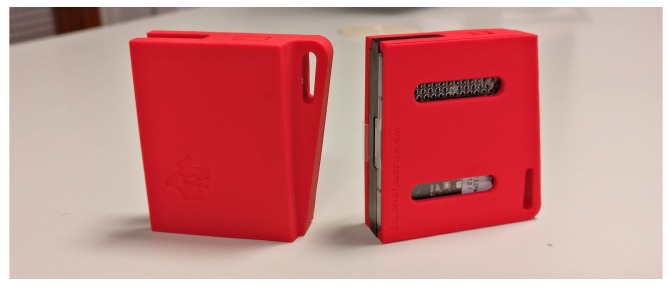
SensorTag from Texas Instrument used in the ePhysio prototype as wearable devices.

The *wearable sensors* have been implemented leveraging the SensorTag platform from Texas Instruments (TI) (TI SensorTag web page: http://www.ti.com/ww/en/wireless_connectivity/sensortag/). SensorTags are prototyping boards specifically designed to rapidly design and implement IoT projects. They are equipped with the CC2650 system-on-chip (TI CC2650 web page: http://www.ti.com/product/CC2650), which has 128 KB of programmable memory and 8 KB of RAM. The chip implements multiple wireless protocols, among them, the Bluetooth low energy and the IEEE 802.15.4 standards, two popular low-power standards for short-range communication. The board, which we show in Figure 7, is specifically designed to be used as a wearable device, i.e., it is battery-powered and has a flexible plastic case with clasps for stripes. Moreover, an optional package is available to integrate a small 1.35-inch screen, which can be used to offer immediate feedback to the user. Finally, the board is equipped with a wide range of sensors which can be used to track, in real time, the patient’s movement, i.e., a nine-axis inertial unit including a three-axial accelerometer, gyroscope, and magnetometer. The platform can be programmed in C using the TI-RTOS software provided by TI or the Contiki Operating System (OS) (ContikiOS web page: https://github.com/contiki-ng/contiki-ng), a popular OS for sensor platforms.

**Figure 8:**
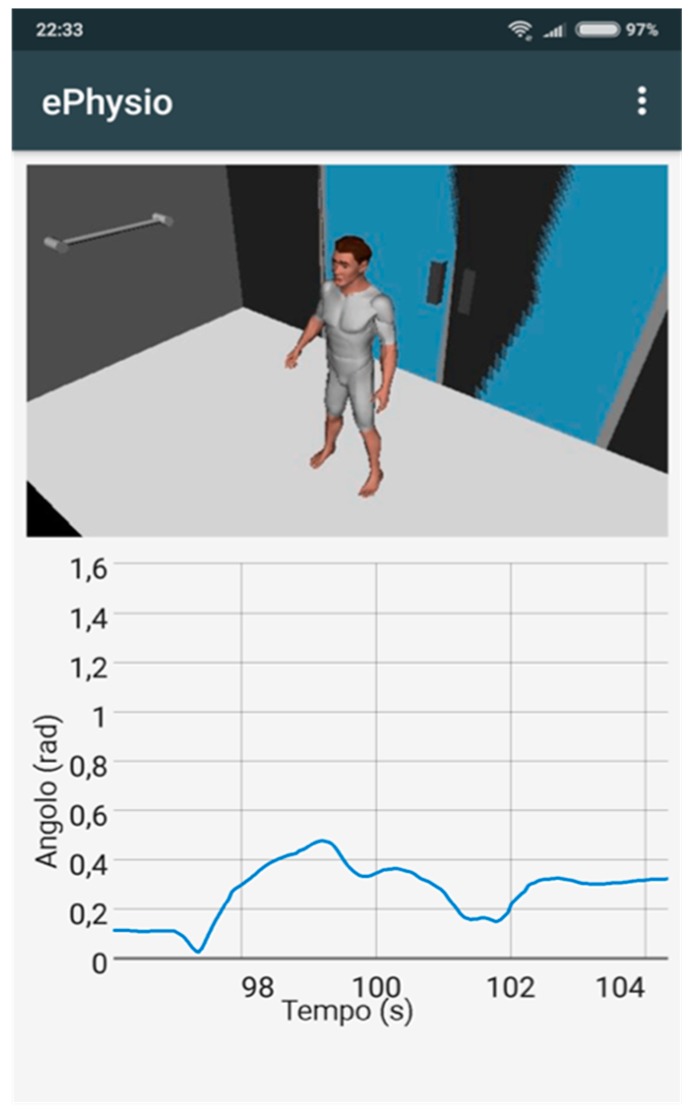
Android application layout.

The *rehabilitation hub* has been implemented as an *Android application*, which can be run on a smartphone or a tablet. The application is used by the user during exercise and carries out two different tasks: (i) to collect data from the sensors and upload them to the cloud backend; (ii) show a user interface that guides the user and provides immediate feedback. To this aim, the Android application connects to the SensorTags via Bluetooth low energy to collect the raw data, e.g., the accelerometer, gyroscope, and magnetometer. The application initially processes the collected data according to the biomechanical model of the exercise. The processed information is enriched with metadata, e.g., patient identifier, exercise identifier, and generation timestamp, and then uploaded to the database of the cloud backend. The layout of the user interface exposed by the application is shown in Figure 8: On the upper part, a video animation illustrating the steps of the exercise to be executed is shown, while on the lower part, a plot of the refined data collected from the sensors, in this case, the Euler angular data, is shown in real time.

**Figure 9:**
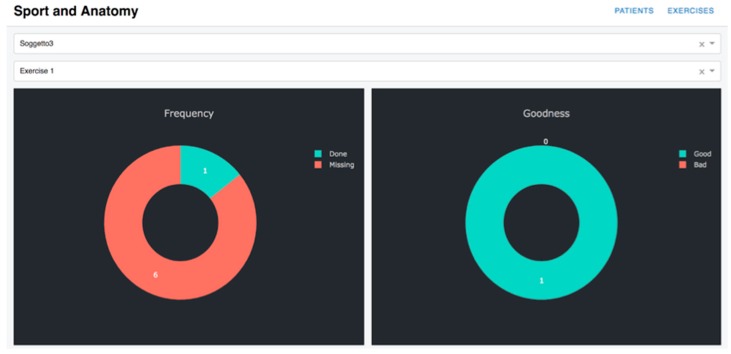
Professional interface, patient overview.

**Figure 10:**
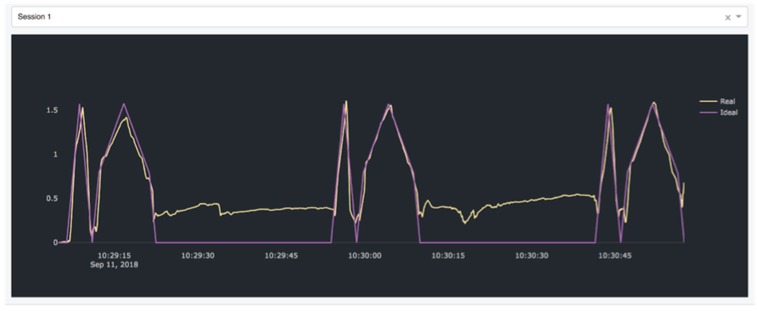
Professional interface, single-exercise detail.

The *cloud system* has been developed as an Ubuntu 16.04 virtual machine hosted on a private cloud platform based on OpenStack, a popular open-source cloud computing platform. The system comprises a MySQL database which stores the data received from the rehabilitation hub and a web interface written in Python. The latter provides an interface for professionals to access all the collected data and analyze them. The interface is implemented using the DASH Pyhton framework (DASH Python framework web page: https://plot.ly/products/dash/) for data visualization. Two exemplary pages of the interface are shown in Figure 9 and Figure 10. In Figure 9, a summary of the data collected from one patient is shown. Through this page, the physician or the physiotherapist can analyze the overall performance of the patient by retrieving the number of exercises performed by each patient (graph on the left) and the number of well-executed exercises (graph on the right) based on an accuracy index evaluated for each exercise by the system. In order to gain an insight on each execution, for each exercise a detail page can be visualized as shown in Figure 10. As can be seen, this page shows the data collected from the sensors, in this case the Euler angle over time. For each exercise, the data from an ideal execution are stored in the system and shown in terms of comparisons to the data collected from patients’ exercises to aid the professional in his/her evaluation. The data from ideal execution are also exploited to evaluate the accuracy index, which measures how a patient’s execution differs from the ideal execution.

**Figure 11:**
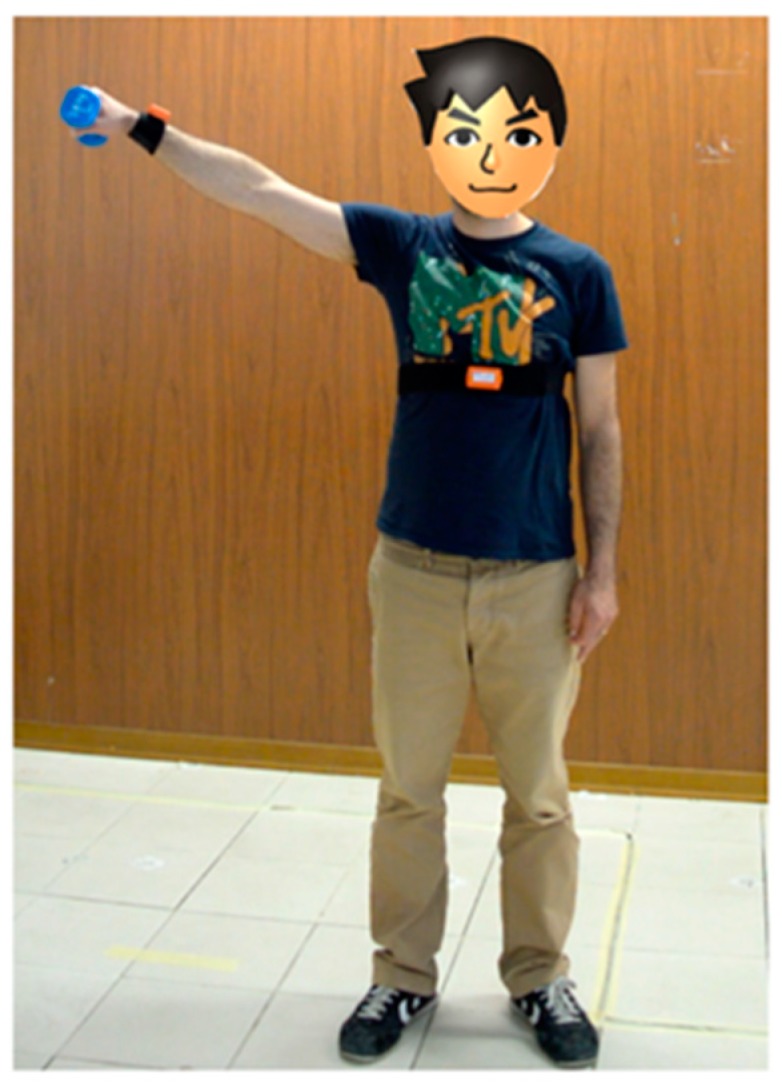
Exercise execution example.

The proof-of-concept deployment has been tested by means of in-lab tests. As the use case, we considered a specific disorder, the shoulder impingement disorder. The disorder is usually treated with anti-inflammatory medications and self-rehabilitation exercises. Each exercise usually involves the patient performing repetitively specific movements of one arm handling a weight, as shown in Figure 11. The exercise is usually prescribed once per day by the physician. The ePhysio system was configured as follows to monitor a patient’s self-rehabilitation. One SensorTag wearable sensor was attached using a textile stripe to the wrist of the arm involved in the exercise; the sensor is programmed using the TI-RTOS software to periodically collect measurements from the accelerometer and gyroscope and send the samples to the hub via Bluetooth. To implement the rehabilitation hub, we exploited an Android tablet placed in proximity of the display realizing the user interface that can guide the patient through the exercise and offer immediate feedback. The Android application is programmed to collect the data from the sensor and pre-process them, calculating the Euler angles, which are eventually uploaded to the cloud system.

During the in-lab tests, the proper functioning of all the ePhysio components was evaluated. To this aim, a healthy volunteer, not affected by the syndrome, was asked to perform a set of rehabilitation sessions. Our tests proved that our implementation can successfully collect real-time data from the patient and store it in the cloud system. In addition to this, the feedback from the volunteer confirmed that the user interface can help the patient by showing the steps of the exercises and a real-time feedback on its execution. Finally, the tests also confirmed the proper functioning of the cloud backend, which was effective in showing the data collected from the various rehabilitation sessions and in evaluating the rehabilitation sessions, by comparing the data of each execution with an ideal execution obtained from a model.

An advanced version of the rehabilitation hub specifically designed to support the multi-user use cases has also been designed and it is currently being implemented. This multi-user rehabilitation hub is based on an embedded system, i.e., a Raspberry PI 3, running the Linux OS. The connection between the sensors and the embedded system is implemented via Bluetooth, while the WiFi interface is exploited to connect the board to the Internet and upload the collected data to the cloud.

This implementation will be used as the basis to implement a multi-user version of the system in a future work. Specifically, we plan to introduce the following functionalities:

- Develop a multi-user rehabilitation hub that can be easily extended. To this aim, we plan to exploit lightweight virtualization technologies, such as Docker [44] and Linux Containers [45], to ensure rapid support of novel therapies and new types of sensors.
- Enrich the cloud platform by enabling the implementation of a machine learning algorithm for data analysis. Such algorithms could help physicians and physiotherapists to analyze the collected data and automatically provide insight and suggestions to assist professionals in their work.

## 6. Conclusions and Future Work

In this paper we present ePhysio, a large-scale and flexible platform for sensor-assisted physiotherapy, which leverages networking and computing tools to provide real-time and ubiquitous monitoring of patients. We first analyzed three possible use cases to draw the set of requirements and then we presented the system architecture. Eventually, we illustrated a proof-of-concept prototype to demonstrate its feasibility by using devices available on the market.

As future work, we aim at extending the current implementation to improve the proof-of-concept towards a mature system that can be adopted on a larger scale. The system will be evaluated by means of real patients with the support of physicians and physiotherapists, to assess the benefits and the overall advantages in a real environment. In addition to this, we also plan to improve the system by introducing novel technologies for the wearable sensors. Specifically, we aim at developing wearable sensors based on a textile technology, which would reduce the impact on the patient and improve the patient’s comfort.
